# Forecasting Alcohol‐Related Liver Disease Mortality Trends in Younger Populations Using Advanced Time‐Series Models: A 1999–2030 Analysis

**DOI:** 10.1002/jgh3.70057

**Published:** 2024-12-03

**Authors:** Hassam Ali, Vinay Jahagirdar, Hanna Blaney, Dushyant Singh Dahiya, Manesh K. Gangwani, Pratik Patel, Umar Hayat, Fouad Jaber, Douglas A. Simonetto, Sanjaya K. Satapathy

**Affiliations:** ^1^ Department of Gastroenterology, Hepatology & Nutrition ECU Health Medical Center/Brody School of Medicine Greenville North Carolina USA; ^2^ Department of Internal Medicine University of Missouri–Kansas City School of Medicine Kansas City Missouri USA; ^3^ Division of Gastroenterology and Hepatology University of Maryland School of Medicine Baltimore Maryland USA; ^4^ Division of Gastroenterology, Hepatology & Motility The University of Kansas School of Medicine Kansas City Kansas USA; ^5^ Department of Medicine University of Toledo Medical Center Toledo Ohio USA; ^6^ Department of Gastroenterology Mather Hospital/Hofstra University Zucker School of Medicine Port Jefferson New York USA; ^7^ Department of Internal Medicine Geisinger Wyoming Valley Medical Center Wilkis Barre Pennsylvania USA; ^8^ Division of Gastroenterology and Hepatology Mayo Clinic Rochester Minnesota USA; ^9^ Division of Hepatology, Sandra Atlas Bass Center for Liver Diseases and Transplantation Donald and Barbara Zucker School of Medicine, Northwell Health Manhasset New York USA

**Keywords:** alcohol‐related disorders, ARIMA, CDC WONDER, epidemiology, forecasting, liver diseases, machine learning, mortality, public health, young adults

## Abstract

**Objective:**

Alcohol‐related liver disease (ALD) has emerged as a significant public health concern, particularly among younger populations. ALD remains the leading cause of alcohol‐attributable deaths. This study aims to forecast ALD mortality trends up to 2030, focusing on individuals under 55 years.

**Methods:**

We utilized data from the CDC WONDER database (1999–2022) to examine ALD‐related deaths, identified by ICD‐10 codes (K70.0–K70.9). Crude mortality rates (CMRs) per 100 000 were analyzed and temporal trends were assessed using annual and average annual percent changes (APC/AAPC) with empirical quantile confidence intervals. An Autoregressive Integrated Moving Average (ARIMA) model was employed to project mortality rates until 2030, validated through time series cross‐validation.

**Results:**

From 1999 to 2022, there were 181 862 ALD‐related deaths among individuals under 55, with mortality rates increasing from 3.9 per 100 000 in 1999 to 9.7 per 100 000 in 2022 (AAPC 4.66%, 95% CI: 3.90%–5.86%). Projections suggest rates will continue to rise, reaching 14.4 per 100 000 by 2030. From 1999 to 2022, the 25–34 age group experienced the highest increase, with an AAPC of 10.27% (95% CI: 9.19%–11.35%), while the 35–44 and 45–54 age groups showed more moderate increases, with AAPCs of 5.03% and 4.38%, respectively. Projections indicate an AAPC of 3.86% for ages 25–34, 3.90% for ages 35–44, and 6.17% for ages 45–54 by 2030.

**Conclusion:**

Forecasts indicate a continued rise in ALD mortality among individuals under 55, necessitating immediate public health strategies to mitigate this trend.

## 1 Introduction

In recent times, alcohol‐related liver disease (ALD) has become a significant public health concern, especially affecting younger demographics. ALD is currently the leading cause of death directly linked to alcohol consumption, highlighting its profound implications [1]. Data from the Centers for Disease Control and Prevention's Wide‐ranging Online Data for Epidemiologic Research system (CDC WONDER) show a concerning increase in alcohol‐related deaths from 2000 to 2020, with a sharp acceleration in recent years [2]. Over the past two decades, there have been notable shifts in alcohol consumption patterns, particularly among women, older individuals, and those from lower socio‐economic backgrounds [3]. These changes emphasize the evolving landscape of alcohol use and its associated health challenges. A global analysis from the Global Burden of Disease (GBD) study between 2000 and 2019 reported that the burden of ALD among individuals aged 15–29 has significantly increased over the past two decades, with notable rises in both prevalence and mortality rates [4]. The study highlighted that ALD prevalence rates increased across all age groups within this demographic, with particularly sharp increases in regions such as Africa and the Eastern Mediterranean. Additionally, another study examining the prevalence of steatotic liver disease (SLD) in a Japanese population identified that alcohol‐associated liver disease remains a critical component of liver disease burden, even in nonobese and lean populations [5].

Our study aimed to predict trends in ALD mortality rates up to 2030, focusing on individuals under 55. Understanding these trends can help better inform public health strategies and interventions to tackle this growing concern.

## 2 Materials and Methods

This study adhered to the Strengthening the Reporting of Observational Studies in Epidemiology (STROBE) guidelines and did not require informed consent or Institutional Review Board (IRB) approval, as it utilized publicly available, deidentified data from the Centers for Disease Control and Prevention's Wide‐Ranging Online Data for Epidemiologic Research (CDC WONDER) database, covering the years 1999–2022 [2].

### 2.1 Study Design and Data Source

This retrospective cross‐sectional study analyzed trends in ALD related mortality among individuals younger than 55 years old. Data were retrieved from the CDC WONDER multiple causes of death database, which includes detailed mortality data for all deaths registered in the United States during the study period. ALD‐related deaths were identified using the International Classification of Diseases, 10th Revision (ICD‐10) codes. ALD‐related mortality was defined as deaths where any of the ICD‐10 codes K70.0‐K70.9 were listed as a cause of death. The study focused on individuals under 55 years old due to emerging evidence that ALD mortality is rising most rapidly within this age group [4, 5]. Recent studies indicate significant increases in alcohol consumption and related mortality among younger adults, necessitating targeted public health interventions [4, 5].

### 2.2 Statistical Analysis

Crude mortality rates (CMRs) per 100 000 population were calculated and age‐adjusted to the 2000 US standard population for comparability across years and demographic groups. Temporal trends in ALD mortality were assessed using Joinpoint regression analysis to identify significant shifts over time, with annual percent changes (APCs) and average annual percent changes (AAPCs) computed along with 95% confidence intervals (CIs).

### 2.3 Forecasting Model

**FIGURE 1:**
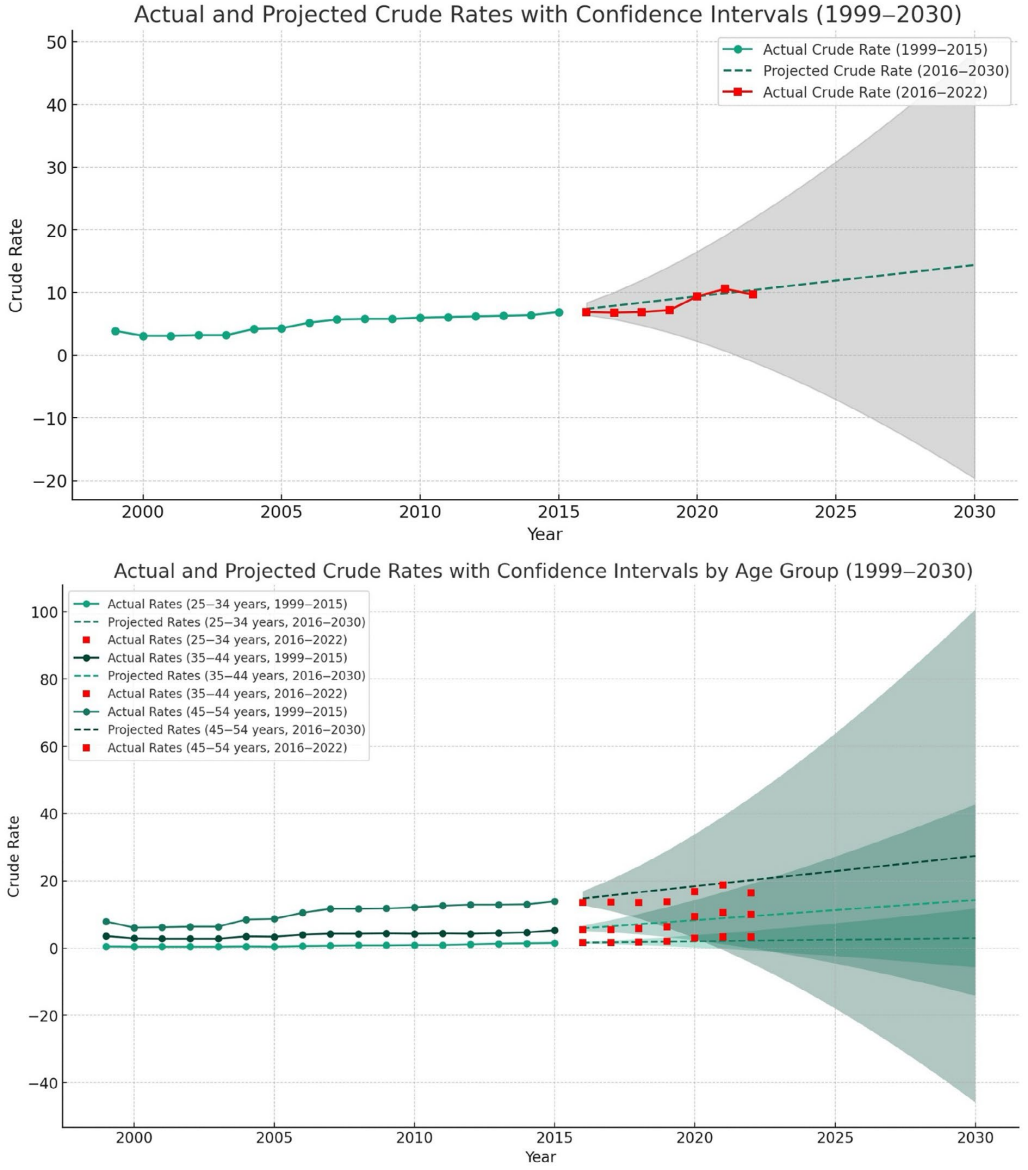
Forecasting crude mortality rates of alcoholic liver disease mortality from 2016 to 2030, total and age group stratified. Crude rate per 100 000. Root mean square error (RMSE) of the model for 2016–2022: 1.04; age group: 25–34 years—RMSE: 0.75; age group: 35–44 years—RMSE: 1.11; age group: 45–54 years—RMSE: 2.56.

To project future ALD mortality trends up to 2030, we employed an Autoregressive Integrated Moving Average (ARIMA) model. The model was trained on data from 1999 to 2015 and validated using data from 2016 to 2022 through time series cross‐validation. Variables analyzed included detailed mortality rates among age groups and sex. Model performance was assessed using Bayesian information criterion (BIC), and root mean square error (RMSE), with projections made for 2023 to 2030 (Figure 1).

## 3 Results

Between 1999 and 2022, there were 181 862 deaths (32.8% females, 84.9% White) related to ALD in patients under 55 years, with rates increasing from 3.9/100000 in 1999 to 9.7/100000 in 2022, showing an AAPC of 4.66% (95% CI: 3.90%–5.86%). The projected rates are expected to rise from 10.9/100000 in 2023 to 14.4/100000 in 2030, showing an AAPC of 4.12% (95% CI: 2.76%–5.01%) (Supporting Information). An ARIMA model was applied using the parameters (*p*, *d*, *q*) of (0, 2, 0), with the BIC values indicating model fit. The overall model for all ages had a BIC of 24.08.

For ages 25–34, there were 13 100 deaths (36.5% females, 79.2% White), which increased from 0.5/100000 in 1999 to 3.3/100000 in 2022, showing an AAPC of 10.27% (95% CI: 9.19%–11.35%). The projected rate will increase from 2.3/100000 in 2023 to 4/100000 in 2030, showing an AAPC of 3.86% (95% CI: 3.71%–4.01%). The ARIMA model for this age group, with parameters (0, 2, 0), achieved the lowest BIC value of −16.75, suggesting the best fit among all models.

For ages 35–44, there were 50 451 deaths (35.1% females, 83.1% White), increasing from 3.6/100000 in 1999 to 10/100000 in 2022, showing an AAPC of 5.03% (95% CI: 3.83%–6.21%). The projected rates are expected to increase from 10.1/100000 in 2023 to 14.3/100000 in 2030, showing an AAPC of 3.90% (95% CI: 1.47%–5.59%). The ARIMA model for this age group had a BIC of 18.70 with parameters (0, 2, 0).

For ages 45–54, there were 118 311 deaths (31.4% females, 86.2% White), which increased from 7.9/100000 in 1999 to 16.4/100000 in 2022, showing an AAPC of 4.38% (95% CI: 3.28%–5.48%). The projected rates are expected to rise from 21.1/100000 in 2023 to 27.4/100000 in 2030, showing an AAPC of 6.17% (95% CI: 5.11%–7.93%) (Supporting Information). The ARIMA model for this age group showed a BIC of 47.07 with parameters (0, 2, 0).

## 4 Discussion

Our research indicates an expected increase in ALD mortality rates up until 2030, with a significant rise among younger cohorts aged 35–44 and 45–54, predominantly affecting Whites and males. Young adults affected by ALD are the demographic experiencing the most rapid increase in liver‐related fatalities.

Recent data support our findings and further underscores the growing public health crisis ALD poses. A 2023 study by the National Institute on Alcohol Abuse and Alcoholism (NIAAA) reports that alcohol consumption increased significantly during the COVID‐19 pandemic, exacerbating existing trends in alcohol‐related health issues [6]. This rise in alcohol consumption during the pandemic has been linked to increased stress, social isolation, and economic uncertainty, factors that disproportionately affect younger adults [7]. Furthermore, recent evidence suggests that alcohol consumption patterns are becoming more harmful, with binge drinking and high intensity drinking episodes on the rise, particularly among young adults [8].

Our findings align with prior research indicating that younger age groups, particularly those aged 25–44, are experiencing the steepest increases in ALD mortality rates [9]. Future projections further support this trend, suggesting that deaths from liver diseases attributable to alcohol consumption will continue rising globally, with a notable increase among males. From 2020 to 2044, the number of deaths from cirrhosis linked to alcohol use is projected to rise significantly, especially in males, while female deaths are expected to remain below the baseline [10]. Although the age‐standardized death rate (ASDR) for cirrhosis is predicted to decrease globally, it may stabilize for a period in males before rising again [10]. In the United States, hospitalization density for alcoholic hepatitis has also increased across racial groups, with a pronounced surge among Hispanics. Prior forecast models suggest continued growth highlighting an urgent need for targeted interventions to address this increasing burden [11].

In addition, there is growing recognition of the role that mental health plays in alcohol use disorders. The intersection of mental health issues and alcohol consumption is critical, as individuals with mental health disorders are more likely to engage in heavy drinking, which can accelerate the progression of ALD [12]. This calls for integrated healthcare approaches that address both mental health and substance use disorders simultaneously.

To the best of our knowledge, our study is among the first to use time‐series predictive models, such as ARIMA, to forecast ALD mortality trends. Machine Learning to develop predictive models may reduce human errors, such as model misspecification [13]. However, it is crucial to recognize that the data used can contain inherent biases, leading to biased algorithm outcomes that may reinforce current inequalities, and acknowledge the risk of misclassification bias in death certificate data [13]. A limitation of the study is the varying BIC values across age groups, suggesting that the model fit is less optimal for older age groups and may require further refinement. Additionally, the inherent uncertainties of long‐term projections, particularly regarding potential shifts in alcohol consumption patterns post‐pandemic, must be acknowledged, as they may significantly influence the accuracy of future mortality trends.

Despite these challenges, the forecasts highlight the critical necessity for specific public health measures to address the growing ALD burden, particularly among younger populations. The pattern of mortality due to ALD has been on an upward trajectory over recent years. It is expected to persist, underscoring the pressing necessity for additional studies to uncover the factors driving these trends. To address the growing ALD burden, integrating screening tools like the WHO's Alcohol Use Disorders Identification Test (AUDIT), brief interventions, and medication‐assisted treatments such as naltrexone or acamprosate within gastroenterology units can help identify and manage at‐risk patients more effectively, improving outcomes.

In conclusion, our study emphasizes the need for comprehensive public health strategies that include early intervention, education, and support services aimed at reducing alcohol consumption and preventing ALD.

## Ethics Statement

Institutional IRB approval was not obtained for this study as the CDC Wonder database is a third‐party de‐identified retrospective database that is publicly accessible.

## Consent

The authors have nothing to report.

## Conflicts of Interest

The authors declare no conflicts of interest.

## Supporting information


Figure S1.

Data S1.
