# Supplementary figures and images for: Forecasting Alcohol‐Related Liver Disease Mortality Trends in Younger Populations Using Advanced Time‐Series Models: A 1999–2030 Analysis
Source: JGH Open. 2024 Dec 3;8(12):e70057. doi: 10.1002/jgh3.70057 (PMC11614748; doi:10.1002/jgh3.70057)

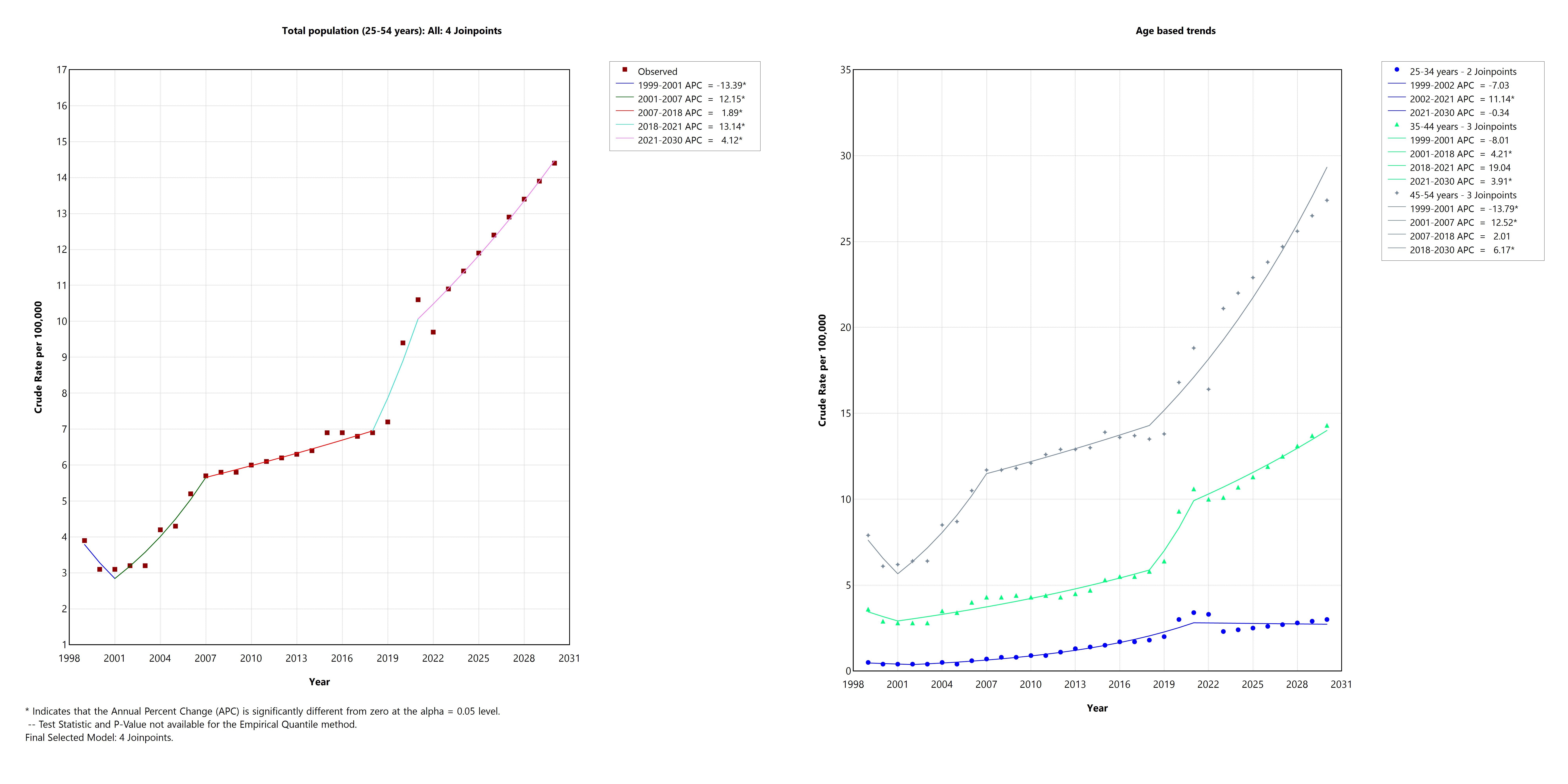

Supplement: Supplementary file 1 — Figure S1. [file JGH3-8-e70057-s002.jpg]
