# Supplementary material for: Forecasting Alcohol‐Related Liver Disease Mortality Trends in Younger Populations Using Advanced Time‐Series Models: A 1999–2030 Analysis
Source: JGH Open. 2024 Dec 3;8(12):e70057. doi: 10.1002/jgh3.70057 (PMC11614748; doi:10.1002/jgh3.70057)
Supplement: Supplementary file 2 — Data S1. [file JGH3-8-e70057-s001.docx]

Supplementary figure 1: Join point trends in ALD-related mortality (Reported 1999-2022) and projected (2023-2030) in the United States, total and stratified by age groups.


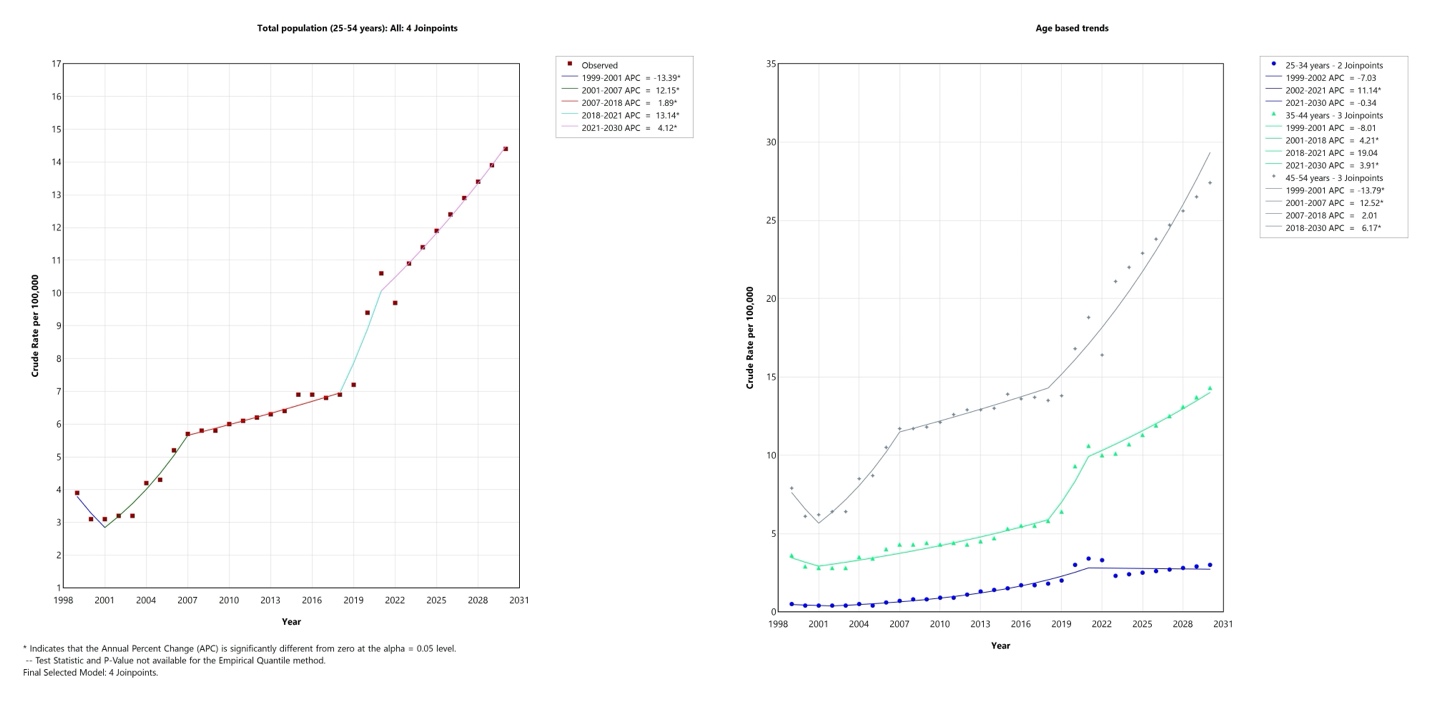


Supplementary Table 1: Reported and projected average annual percent changes (AAPC) for Alcoholic related liver disease mortality in patients younger than 55 years

| **Range** | **AAPC** | **Lower CI** | **Upper CI** |
| --- | --- | --- | --- |
| 1999-2016 | 3.40* | 2.84 | 4.04 |
| 2017-2022 | 8.96* | 7.35 | 10.64 |
| 2023-2030 | 4.11* | 2.75 | 5.00 |

Supplementary table 2: Reported and projected crude mortality rates /100,000 for Alcoholic related liver disease mortality in patients younger than 55 years

| Year | Crude Rate | Crude Rate Lower 95% Confidence Interval | Crude Rate Upper 95% Confidence Interval |
| --- | --- | --- | --- |
| 1999 | 3.9 | 3.8 | 4 |
| 2000 | 3.1 | 3 | 3.2 |
| 2001 | 3.1 | 3 | 3.2 |
| 2002 | 3.2 | 3.1 | 3.3 |
| 2003 | 3.2 | 3.1 | 3.3 |
| 2004 | 4.2 | 4.1 | 4.3 |
| 2005 | 4.3 | 4.1 | 4.4 |
| 2006 | 5.2 | 5.1 | 5.3 |
| 2007 | 5.7 | 5.6 | 5.9 |
| 2008 | 5.8 | 5.6 | 5.9 |
| 2009 | 5.8 | 5.7 | 6 |
| 2010 | 6 | 5.9 | 6.1 |
| 2011 | 6.1 | 6 | 6.3 |
| 2012 | 6.2 | 6.1 | 6.4 |
| 2013 | 6.3 | 6.2 | 6.4 |
| 2014 | 6.4 | 6.2 | 6.5 |
| 2015 | 6.9 | 6.8 | 7 |
| 2016 | 6.9 | 6.7 | 7 |
| 2017 | 6.8 | 6.7 | 7 |
| 2018 | 6.9 | 6.7 | 7 |
| 2019 | 7.2 | 7.1 | 7.3 |
| 2020 | 9.4 | 9.2 | 9.6 |
| 2021 | 10.6 | 10.5 | 10.8 |
| 2022 | 9.7 | 9.5 | 9.8 |
| **2023** | **10.9** | **-2.91** | **24.71** |
| **2024** | **11.4** | **-4.92** | **27.72** |
| **2025** | **11.9** | **-7.07** | **30.87** |
| **2026** | **12.4** | **-9.35** | **34.15** |
| **2027** | **12.9** | **-11.75** | **37.55** |
| **2028** | **13.4** | **-14.27** | **41.07** |
| **2029** | **13.9** | **-16.9** | **44.7** |
| **2030** | **14.4** | **-19.65** | **48.45** |

Bold: Projected.

Supplementary table 3: Reported and projected crude mortality rates /100,000 for Alcoholic related liver disease mortality in patients younger than 55 years, stratified by age groups.

| Ten-Year Age Groups | Year | Crude Rate | Crude Rate Lower 95% Confidence Interval | Crude Rate Upper 95% Confidence Interval |
| --- | --- | --- | --- | --- |
| 25-34 years | 1999 | 0.5 | 0.4 | 0.6 |
| 25-34 years | 2000 | 0.4 | 0.3 | 0.5 |
| 25-34 years | 2001 | 0.4 | 0.3 | 0.5 |
| 25-34 years | 2002 | 0.4 | 0.3 | 0.4 |
| 25-34 years | 2003 | 0.4 | 0.3 | 0.4 |
| 25-34 years | 2004 | 0.5 | 0.4 | 0.5 |
| 25-34 years | 2005 | 0.4 | 0.4 | 0.5 |
| 25-34 years | 2006 | 0.6 | 0.5 | 0.7 |
| 25-34 years | 2007 | 0.7 | 0.7 | 0.8 |
| 25-34 years | 2008 | 0.8 | 0.7 | 0.9 |
| 25-34 years | 2009 | 0.8 | 0.7 | 0.9 |
| 25-34 years | 2010 | 0.9 | 0.8 | 1 |
| 25-34 years | 2011 | 0.9 | 0.8 | 1 |
| 25-34 years | 2012 | 1.1 | 1 | 1.2 |
| 25-34 years | 2013 | 1.3 | 1.2 | 1.4 |
| 25-34 years | 2014 | 1.4 | 1.2 | 1.5 |
| 25-34 years | 2015 | 1.5 | 1.4 | 1.7 |
| 25-34 years | 2016 | 1.7 | 1.6 | 1.8 |
| 25-34 years | 2017 | 1.7 | 1.5 | 1.8 |
| 25-34 years | 2018 | 1.8 | 1.7 | 1.9 |
| 25-34 years | 2019 | 2 | 1.9 | 2.2 |
| 25-34 years | 2020 | 3 | 2.8 | 3.1 |
| 25-34 years | 2021 | 3.4 | 3.2 | 3.5 |
| 25-34 years | 2022 | 3.3 | 3.1 | 3.5 |
| 25-34 years | 2023 | 2.3 | -1.24 | 5.84 |
| 25-34 years | 2024 | 2.4 | -1.79 | 6.59 |
| 25-34 years | 2025 | 2.5 | -2.36 | 7.36 |
| 25-34 years | 2026 | 2.6 | -2.98 | 8.18 |
| 25-34 years | 2027 | 2.7 | -3.62 | 9.02 |
| 25-34 years | 2028 | 2.8 | -4.29 | 9.89 |
| 25-34 years | 2029 | 2.9 | -5 | 10.8 |
| 25-34 years | 2030 | 3 | -5.73 | 11.73 |
| 35-44 years | 1999 | 3.6 | 3.5 | 3.8 |
| 35-44 years | 2000 | 2.9 | 2.8 | 3.1 |
| 35-44 years | 2001 | 2.8 | 2.6 | 2.9 |
| 35-44 years | 2002 | 2.8 | 2.6 | 3 |
| 35-44 years | 2003 | 2.8 | 2.6 | 2.9 |
| 35-44 years | 2004 | 3.5 | 3.3 | 3.7 |
| 35-44 years | 2005 | 3.4 | 3.2 | 3.6 |
| 35-44 years | 2006 | 4 | 3.8 | 4.2 |
| 35-44 years | 2007 | 4.3 | 4.1 | 4.5 |
| 35-44 years | 2008 | 4.3 | 4.1 | 4.5 |
| 35-44 years | 2009 | 4.4 | 4.2 | 4.6 |
| 35-44 years | 2010 | 4.3 | 4.1 | 4.5 |
| 35-44 years | 2011 | 4.4 | 4.2 | 4.6 |
| 35-44 years | 2012 | 4.3 | 4.1 | 4.5 |
| 35-44 years | 2013 | 4.5 | 4.3 | 4.7 |
| 35-44 years | 2014 | 4.7 | 4.5 | 4.9 |
| 35-44 years | 2015 | 5.3 | 5.1 | 5.5 |
| 35-44 years | 2016 | 5.5 | 5.2 | 5.7 |
| 35-44 years | 2017 | 5.5 | 5.3 | 5.8 |
| 35-44 years | 2018 | 5.8 | 5.6 | 6.1 |
| 35-44 years | 2019 | 6.4 | 6.1 | 6.6 |
| 35-44 years | 2020 | 9.3 | 9 | 9.6 |
| 35-44 years | 2021 | 10.6 | 10.3 | 10.9 |
| 35-44 years | 2022 | 10 | 9.7 | 10.3 |
| 35-44 years | 2023 | 10.1 | -1.44 | 21.64 |
| 35-44 years | 2024 | 10.7 | -2.94 | 24.34 |
| 35-44 years | 2025 | 11.3 | -4.56 | 27.16 |
| 35-44 years | 2026 | 11.9 | -6.28 | 30.08 |
| 35-44 years | 2027 | 12.5 | -8.1 | 33.1 |
| 35-44 years | 2028 | 13.1 | -10.03 | 36.23 |
| 35-44 years | 2029 | 13.7 | -12.05 | 39.45 |
| 35-44 years | 2030 | 14.3 | -14.16 | 42.76 |
| 45-54 years | 1999 | 7.9 | 7.6 | 8.2 |
| 45-54 years | 2000 | 6.1 | 5.8 | 6.3 |
| 45-54 years | 2001 | 6.2 | 6 | 6.5 |
| 45-54 years | 2002 | 6.4 | 6.2 | 6.7 |
| 45-54 years | 2003 | 6.4 | 6.2 | 6.7 |
| 45-54 years | 2004 | 8.5 | 8.3 | 8.8 |
| 45-54 years | 2005 | 8.7 | 8.4 | 9 |
| 45-54 years | 2006 | 10.5 | 10.2 | 10.8 |
| 45-54 years | 2007 | 11.7 | 11.3 | 12 |
| 45-54 years | 2008 | 11.7 | 11.3 | 12 |
| 45-54 years | 2009 | 11.8 | 11.5 | 12.1 |
| 45-54 years | 2010 | 12.1 | 11.8 | 12.5 |
| 45-54 years | 2011 | 12.6 | 12.2 | 12.9 |
| 45-54 years | 2012 | 12.9 | 12.6 | 13.2 |
| 45-54 years | 2013 | 12.9 | 12.6 | 13.2 |
| 45-54 years | 2014 | 13 | 12.7 | 13.3 |
| 45-54 years | 2015 | 13.9 | 13.5 | 14.2 |
| 45-54 years | 2016 | 13.6 | 13.3 | 14 |
| 45-54 years | 2017 | 13.7 | 13.3 | 14 |
| 45-54 years | 2018 | 13.5 | 13.1 | 13.8 |
| 45-54 years | 2019 | 13.8 | 13.5 | 14.2 |
| 45-54 years | 2020 | 16.8 | 16.4 | 17.2 |
| 45-54 years | 2021 | 18.8 | 18.3 | 19.2 |
| 45-54 years | 2022 | 16.4 | 16 | 16.8 |
| 45-54 years | 2023 | 21.1 | -8.62 | 50.82 |
| 45-54 years | 2024 | 22 | -13.13 | 57.13 |
| 45-54 years | 2025 | 22.9 | -17.93 | 63.73 |
| 45-54 years | 2026 | 23.8 | -23.01 | 70.61 |
| 45-54 years | 2027 | 24.7 | -28.36 | 77.76 |
| 45-54 years | 2028 | 25.6 | -33.95 | 85.15 |
| 45-54 years | 2029 | 26.5 | -39.8 | 92.8 |
| 45-54 years | 2030 | 27.4 | -45.88 | 100.68 |

Supplementary table 4: Reported and projected crude mortality rates /100,000 for Alcoholic related liver disease mortality in patients younger than 55 years, stratified by sex.

|  | Year | Crude Rate | Crude Rate Lower 95% Confidence Interval | Crude Rate Upper 95% Confidence Interval |
| --- | --- | --- | --- | --- |
| Females | 1999 | 2 | 1.9 | 2.1 |
|  | 2000 | 1.7 | 1.6 | 1.8 |
|  | 2001 | 1.8 | 1.7 | 1.9 |
|  | 2002 | 1.9 | 1.8 | 2 |
|  | 2003 | 1.8 | 1.7 | 1.9 |
|  | 2004 | 2.4 | 2.3 | 2.5 |
|  | 2005 | 2.5 | 2.4 | 2.6 |
|  | 2006 | 3.1 | 2.9 | 3.2 |
|  | 2007 | 3.4 | 3.2 | 3.5 |
|  | 2008 | 3.5 | 3.3 | 3.6 |
|  | 2009 | 3.7 | 3.5 | 3.8 |
|  | 2010 | 3.9 | 3.7 | 4 |
|  | 2011 | 4.2 | 4 | 4.3 |
|  | 2012 | 4.1 | 3.9 | 4.2 |
|  | 2013 | 4.1 | 4 | 4.3 |
|  | 2014 | 4.4 | 4.2 | 4.5 |
|  | 2015 | 4.8 | 4.6 | 5 |
|  | 2016 | 4.9 | 4.7 | 5.1 |
|  | 2017 | 4.8 | 4.6 | 5 |
|  | 2018 | 4.9 | 4.7 | 5 |
|  | 2019 | 5.1 | 4.9 | 5.3 |
|  | 2020 | 6.7 | 6.5 | 6.9 |
|  | 2021 | 7.4 | 7.1 | 7.6 |
|  | 2022 | 6.8 | 6.6 | 7 |
| Male | 1999 | 5.8 | 5.6 | 5.9 |
|  | 2000 | 4.5 | 4.3 | 4.7 |
|  | 2001 | 4.5 | 4.3 | 4.6 |
|  | 2002 | 4.5 | 4.3 | 4.7 |
|  | 2003 | 4.7 | 4.5 | 4.9 |
|  | 2004 | 6.1 | 5.9 | 6.3 |
|  | 2005 | 6.1 | 5.9 | 6.3 |
|  | 2006 | 7.3 | 7.1 | 7.6 |
|  | 2007 | 8.1 | 7.9 | 8.3 |
|  | 2008 | 8.1 | 7.9 | 8.3 |
|  | 2009 | 8 | 7.8 | 8.3 |
|  | 2010 | 8.1 | 7.9 | 8.3 |
|  | 2011 | 8.1 | 7.9 | 8.4 |
|  | 2012 | 8.4 | 8.2 | 8.6 |
|  | 2013 | 8.5 | 8.2 | 8.7 |
|  | 2014 | 8.4 | 8.2 | 8.6 |
|  | 2015 | 9 | 8.8 | 9.3 |
|  | 2016 | 8.9 | 8.6 | 9.1 |
|  | 2017 | 8.9 | 8.6 | 9.1 |
|  | 2018 | 8.9 | 8.7 | 9.1 |
|  | 2019 | 9.3 | 9.1 | 9.6 |
|  | 2020 | 12 | 11.8 | 12.3 |
|  | 2021 | 13.9 | 13.6 | 14.1 |
|  | 2022 | 12.6 | 12.3 | 12.8 |
